# Tibial shaft fractures in football players

**DOI:** 10.1186/1749-799X-2-11

**Published:** 2007-06-13

**Authors:** Winston R Chang, Zain Kapasi, Susan Daisley, William J Leach

**Affiliations:** 1 Department of Trauma and Orthopaedics, Western Infirmary, Glasgow, UK

## Abstract

**Background:**

Football is officially the most popular sport in the world. In the UK, 10% of the adult population play football at least once a year. Despite this, there are few papers in the literature on tibial diaphyseal fractures in this sporting group. In addition, conflicting views on the nature of this injury exist. The purpose of this paper is to compare our experience of tibial shaft football fractures with the little available literature and identify any similarities and differences.

**Methods and Results:**

A retrospective study of all tibial football fractures that presented to a teaching hospital was undertaken over a 5 year period from 1997 to 2001. There were 244 tibial fractures treated. 24 (9.8%) of these were football related. All patients were male with a mean age of 23 years (range 15 to 29) and shin guards were worn in 95.8% of cases. 11/24 (45.8%) were treated conservatively, 11/24 (45.8%) by Grosse Kemp intramedullary nail and 2/24 (8.3%) with plating. A difference in union times was noted, conservative 19 weeks compared to operative group 23.9 weeks (p < 0.05). Return to activity was also different in the two groups, conservative 27.6 weeks versus operative 23.3 weeks (p < 0.05). The most common fracture pattern was AO Type 42A3 in 14/24 (58.3%). A high number 19/24 (79.2%) were simple transverse or short oblique fractures. There was a low non-union rate 1/24 (4.2%) and absence of any open injury in our series.

**Conclusion:**

Our series compared similarly with the few reports available in the literature. However, a striking finding noted by the authors was a drop in the incidence of tibial shaft football fractures. It is likely that this is a reflection of recent compulsory FIFA regulations on shinguards as well as improvements in the design over the past decade since its introduction.

## Background

Football is officially the most popular sport in the world. The Fédération Internationale de Football Association (FIFA) estimates that there are 250 million licensed players in 204 countries with 1% participation at professional level [1]. In the UK, it is estimated that about 10% of the adult population play football at least once a year [2]. It is therefore of considerable importance to the social fabric of society especially in Glasgow where there are two derby teams. Despite this, there are very few good papers in the literature on the epidemiology of tibial shaft fractures in this sporting group [3,4]. In addition, there are conflicting views in the literature. One study described football-related tibial diaphyseal fractures as low-velocity injuries, and very rarely associated with severe soft tissue damage [4]. Other studies [5,6], suggest that lower leg fractures in footballers are serious and potentially high-energy injuries. Nevertheless an interesting observation is that studies carried out in the late 80's and early 90's had relatively higher numbers [3,4], as compared to recent studies where numbers are noted to be relatively lower [5,6]. This may be a subtle hint of a decrease in the incidence of these fractures.

We herein present our experience over a five year period of tibial shaft football fractures in an attempt to identify similarities and differences with the little available literature on this common sport.

## Methods

**Figure 1:**
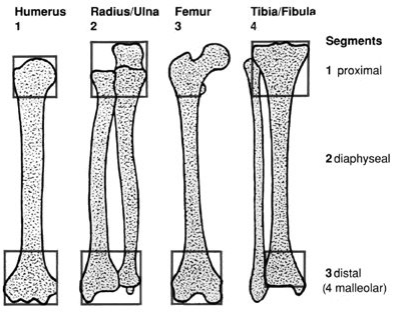
The four long bones and their segments [12].

The casenotes for all tibial fractures treated between 1997 and 2001 inclusive at the Western Infirmary, Glasgow were identified using the hospital "Patient Administration System". Of these, those arising as a result of a football injury were retrospectively studied. Their casenotes and radiographs were reviewed to establish the mechanism of injury, type of fracture, treatment modality and their outcome. A standardized data extraction proforma was used to compile this data. All radiographs were classified according to the AO/ASIF classification system (table 1). The anatomic location of a fracture is designated by two numbers, one for the bone and one for its segment. Each long bone has three segments: the proximal, the diaphyseal, and the distal segment (figure 1). The malleolar segment is an exception and is classified as the fourth segment of the tibia/fibula (44-). Tibial diaphyseal fractures were therefore defined as AO Type 42 diaphyseal fractures excluding the proximal and distal metaphyseal regions (figure 1). The radiographs were analyzed by a single person (third author) to eliminate the possibility of any interobserver variability. Follow up data were collected by telephone questionnaire.

**Table 1:** AO/ASIF classification of tibia shaft fractures [4]

Type	Fracture	Subclassification
A	Simple	A1 – spiral
		A2 – oblique
		A3 – transverse
B	Wedge	B1 – spiral wedge
		B2 – bending wedge
		B3 – fragmented wedge
C	Complex	C1 – spiral
		C2 – segmental
		C3 – irregular

AO/ASIF, Arbeitsgemeinschaft Osteosynthesefragen/Association for the study of Internal Fixation.

Fracture union was defined as pain-free weight-bearing without support; and bridging callus seen on 2 radiographs taken at 90 degrees to each other. Delayed union and non-union were defined as absence of callus on radiographs at 4 and 6 months respectively [6].

The independent t-test was used for statistical analysis of the results and a p value of less than 0.05 was considered significant.

## Results

In the 5 years period from 1997 to 2001, there were 244 tibial fractures treated. 24 (9.8%) of these were football related. Of these, 3 were professional soccer players and 21 amateurs. All patients were male with a mean age of 23 years (range 15 to 29). The right tibia was fractured in 91.7% (22 patients) and the left in 8.3%. The mechanism of injury in almost all cases, 23/24 (95.8%), involved direct contact. Shinguards were also worn in 95.8% of cases. 14 cases (58.3%) occurred on a weekend whilst 10 cases (41.7%) occurred on a weekday.

### Fracture classification

Both tibia and fibula were fractured in 22/24 (91.7%) cases whilst 2 (8.3%) involved the tibia only. There were no open injuries. The fracture types by AO classification are summarized in Table 2.

**Table 2:** Summary of tibial shaft football fractures according to AO type.

**Simple type**	**Number**	**Wedge type**	**Number**
A1	3	B1	1
A2	5	B2	1
A3	14	B3	0
Total	22	Total	2

### Mode of Treatment

11/24 (45.8%) patients were treated conservatively. A standard regimen was followed in the conservative group which consisted of a non-weight bearing above knee plaster for 8 weeks followed by a Sarmiento cast with partial weight bearing until union occurred. The average in-patient time was 2.4 days (range 2–4 days). Mean time to fracture union was 19 weeks (standard deviation 4.05 weeks). The mean length of time taken to return to activity/training was 27.6 weeks (standard deviation 4.54 weeks).

The remaining patients, 13/24 (54.2%), were treated operatively with 11/24 (45.8%), treated with a Grosse Kemp intramedullary nail according to the manufacturer's instructions with primary locking in all cases. The remaining 2 cases were treated with open reduction and internal fixation using a DCP plate and screws (closed 42A1); and interfragmentary screws (43A1) respectively. All patients who underwent intramedullary nailing were allowed partial weight bearing for the first 6 weeks, followed by full weight bearing as tolerated until union. The mean time from admission to fixation was 20.9 hrs (range 3 to 39 hrs). Inpatient time averaged 3.7 days (range 2 to 6 days). Mean time to union was 23.9 weeks (standard deviation 3.99 weeks). Despite this however, the average time to return to training/activity was slightly quicker at 23.3 weeks (standard deviation 6.46 weeks).

The differences in time to union (p < 0.005) and return to activity (p < 0.05) between those treated conservatively and operatively were found to be significant

### Complications

In total, there were 10 cases with complications summarized in Table 4. Loss of position was the most common complication amongst the conservatively treated group 4/11 (36.4%). However, in one case the fibula was intact, whilst another was a professional football player. All were converted to an intramedullary nail. Only one fracture in the conservative group did not unite and required open reduction and internal plating with bone grafting and fibulectomy and was lost to follow up.

**Table 4:** Summary of complications of tibial shaft football fractures according to treatment method and their outcomes.

**AO Class**	**Treatment**	**Complication**	**Outcome**
Closed 42A2	Cast	Position slipped at day 12	IM nail. Nail was removed at 13 months because of anterior knee pain.
Closed 42A3 (fibula intact)	Cast	Position slipped at day 9	IM nail
Closed 42A2	Cast	Position slipped at day 28	IMnail
Closed 42A3	Cast	Position slipped at 8	IM nail
Closed 42A3	Cast	Non-union at 5 months	Underwent bone grafting and plating plus fibulectomy. Subsequently lost to follow-up.
Closed 42A3	IM nail	Anterior knee pain	Nail removed at 29 months
Closed 42A3	IM nail	Delayed union	Dynamisation at 24 weeks
Closed 42A3	IM nail	Anterior knee pain	Nail removed at 24 weeks
Closed 42B2	IM nail (at 23 hrs)	Fat embolism	Admitted to Intensive Care for 2 days then discharged on day 8 post-op. Fracture subsequently united.
Closed 42A2	IM nail	Anterior and medial knee pain.	Self-discharged from clinic and lost to follow-up.

Amongst the operatively treated group, anterior knee pain was the most common complication, 3/13 (23.1%). One patient (42B2) whose fixation interval was 23 hours had respiratory complications related to fat embolism that required supportive care and observation in the intensive care unit for 2 days. Dynamisation was carried out in one case that subsequently united at 9 months.

No patients in either group developed compartment syndrome.

## Discussion

**Figure 2:**
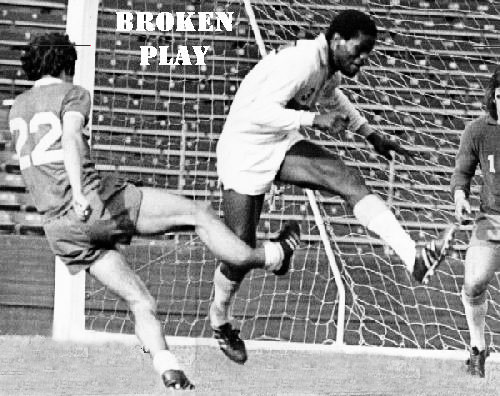
This picture demonstrates an example of the typical mechanism of injury involving a direct blow that results in a tibial fracture.

Our experience shows notable similarities and differences when compared with the few reports available in the literature on such a widely played sport [5,3,4]. The most common fracture pattern was the transverse AO Type 42A3 in 14/24 (58.3%). A high number 19/24 (79.2%) were simple transverse or short oblique fractures. This is consistent with the mechanism of injury involving a direct blow (figure 2) and low-velocity as well as with previous attempts to define the "footballer's fracture". Cattermole et al [3] reported a direct blow in 95% of cases and our finding mirrors this (95.8%). In fact, further support for this is shown by the low non-union rate 1/24 (4.2%) and the absence of an open injury in our series. The Edinburgh study found that 95.4% were closed injuries and of these 90% were Tscherne type 0 or 1 [4]. Delayed union and non-union were also found to be low both by the Leicester study and Lenehan et al quoted a 2% incidence [7]. The latter simply reflects the 'personality' of the tibial fracture as coined by Nicol in 1964 [8] as well as the low mean age of the study population.

Union times were noted to be quicker in the conservative group (p < 0.005). This may be a reflection of the self-selecting bias of those fractures treated by intramedullary nailing. Whilst the majority of fractures in both treatment groups were A2 and A3, of these, the more displaced and hence more severe ones would have been treated by nailing.resulting in the longer healing time. This again is a reflection of the personality of each fracture. On the other hand, return to activity was earlier in the operative group (p < 0.05), a well recognized fact [9]. It facilitates earlier mobilization, hence preservation of muscle mass and prevention of joint stiffness. which would otherwise be present after treatment in a cast.

A striking finding in our study is the much lower incidence of football fractures amongst all tibial shaft fractures 24/244 (9.8%). The Edinburgh study period was from 1988 to 1990 with an incidence of 24.7% [4] whilst, Leeds looked at the period from 1990 to 1994 and quoted an incidence of 17.6% [6]. This corresponded to an introduction of shinguards by FIFA in 1990 as part of the compulsory basic equipment of a player [10]. Shin guards protect by spreading loads over wider areas of the skin. The force of the initial impact is reduced as peak pressure is dampened down. Over the past decade there have been improvements in shinguards since its introduction. Francisco et al tested 23 commercially available shinguards and found that they reduced force by 11% to 17% and strain by 45% to 51% compared with the unguarded leg [11]. The introduction of shinguards with its design improvements may explain the lower incidence in our most recent of all previous study periods (1997 to 2001). In fact, shinguards were worn in 95.8% of cases which testified to its current widespread usage.

## Conclusion

The nature and pattern of tibial shaft football fractures in our series compared similarly with previously published series. One exception noted however, was a decreasing trend in the incidence of tibial football fractures. A possible explanation for this may have been the introduction of shin-pads and improvement in their designs.

## Competing interests

The authors declare that they have no competing interests. No benefits in any form have been received or will be received from a commercial party related directly or indirectly to the subject of this article.

## Authors' contributions

The first three authors contributed to the planning, execution and completion of the project. The article was written up by the first author with advice and guidance from the fourth (senior) author who conceptualized the topic of this article. All authors read and approved the manuscript.

**Table 3:** Treatments used according to AO fracture type.

**AO fracture type**	**Cast**	**Intramedullary nail**	**Plate and screws**
A1	1	0	2
A2	2	3	0
A3	7	7	0
B1	1	0	0
B2	0	1	0
Total	11	11	2
